# Etymologia: *Rickettsia *

**DOI:** 10.3201/eid1109.ET1109

**Published:** 2005-09

**Keywords:** Etymology, etmologia, rickettsia, Howard Taylor Ricketts

## [rĭ-ket′se-ə]

Genus of gram-negative, rod-shaped or coccoid bacteria that are transmitted by lice, fleas, ticks and mites. Named after American pathologist Howard Taylor Ricketts; despite the similar name, Rickettsia spp. do not cause rickets (from the Greek rhakhis, “spine”), a disorder of bone development caused by vitamin deficiency. 

Source: Dorland’s illustrated medical dictionary.30th ed. Philadelphia: Saunders, 2003.

